# Infective Endocarditis Complicated by Septic Embolization in a Patient With Barlow's Disease: A Case Report and Literature Review

**DOI:** 10.7759/cureus.87702

**Published:** 2025-07-10

**Authors:** Aoumar G Chamma, Wendy E Saliba, Maroun Soueidy

**Affiliations:** 1 Cardiology, University of Balamand, Beirut, LBN; 2 Interventional Cardiology, Mount Lebanon Hospital, Beirut, LBN; 3 Interventional Cardiology, Sacre Coeur Hospital, Beirut, LBN

**Keywords:** barlow’s disease, cerebral septic emboli, mitral valve prolapse, transthoracic echocardiogram, valvular endocarditis

## Abstract

Infective endocarditis (IE) is a severe cardiac infection with significant morbidity and mortality. Prompt diagnosis and management, often guided by the Duke’s criteria, are essential for optimal outcomes. We report a case of mitral valve IE caused by methicillin-sensitive Staphylococcus aureus (MSSA) complicated by ischemic stroke and recurrent mitral valve dysfunction in a previously healthy 35-year-old male with unrevealed Barlow's disease. This case highlights the crucial role of timely surgical intervention and multidisciplinary approach in care including cardiology, infectious diseases, and cardiothoracic surgery.

## Introduction

Infective endocarditis (IE), defined as endocardial surface infection, typically presents with nonspecific symptoms that include fever, malaise, weight loss, night sweats and embolic complications [1]. The modified Duke’s criteria include clinical signs, confirmatory microbiological cultures and echocardiographic evidence [2]. They serve as the primary diagnostic tool for this condition. Staphylococcus aureus remains the primary cause of infective endocarditis and may lead to severe clinical progression along with multiple embolic complications. Furthermore, patients who have structural heart diseases including mitral valve prolapse and congenital heart anomalies face elevated risks for infective endocarditis due to the turbulent blood flow that generates optimal conditions for bacterial colonization [3]. A specific type of mitral valve prolapse, known as Barlow’s disease, increases the risk for infective endocarditis due to its thickened mitral leaflets and redundant tissue that make it more susceptible to bacterial adherence and vegetation growth [4]. Staphylococcal species linked to dental procedures warrant adequate clinical monitoring due to the atypical presentation of methicillin-sensitive Staphylococcus aureus (MSSA). This should raise high clinical suspicion in patients with structurally abnormal heart valves [5].

Barlow’s disease is a specific, severe form of mitral valve prolapse (MVP) that presents with widespread myxomatous leaflet thickening and redundant tissue, along with bileaflet or multiscallop prolapse. Also, it is associated with a dilated flattened annulus and generalized chordal elongation [6]. The majority of prolapsing valves do not have Barlow’s disease. However, every Barlow valve will prolapse. This distinction matters because Barlow valves develop severe regurgitation at a faster rate and increase the risk of arrhythmias [6].

Epidemiological data show that Barlow’s morphology of valves represents about 40% of primary degenerative MVP cases found in surgical studies, although its actual prevalence in the general population remains lower at 0.2-0.4% [7]. The large size of redundant leaflets, together with excess chordal tissue, results in a bigger bacterial seeding area that leads to bulky mobile vegetations which increase both infective endocarditis risk and systemic embolization risk compared to simple MVP [8]. The classical link between dental manipulations and viridans group streptococci exists but Staphylococcus aureus, particularly methicillin-sensitive strains that reside on skin or mucosa, can enter the bloodstream through dental extractions when aseptic procedures fail [9]. Our patient underwent a molar extraction without prophylaxis, which probably served as the entry point for the infection.

## Case presentation

Clinical history

A previously healthy 35-year-old male was admitted with a one-week history of fever of unknown origin. One week prior to presentation, he had been admitted to another hospital complaining of persistent fever. He was treated with third-generation cephalosporins without clinical improvement.

Laboratory findings

Blood tests revealed bicytopenia with WBC 11× 10^9/L, hemoglobin of 9.5 g/dL, platelets at 54 × 10³/μL, and elevated CRP reaching 150 mg/L. The oncology team, concerned for an underlying malignancy, ordered a bone marrow biopsy which was performed and showed no abnormal findings.

Imaging and transthoracic echocardiography

**Figure 1:**
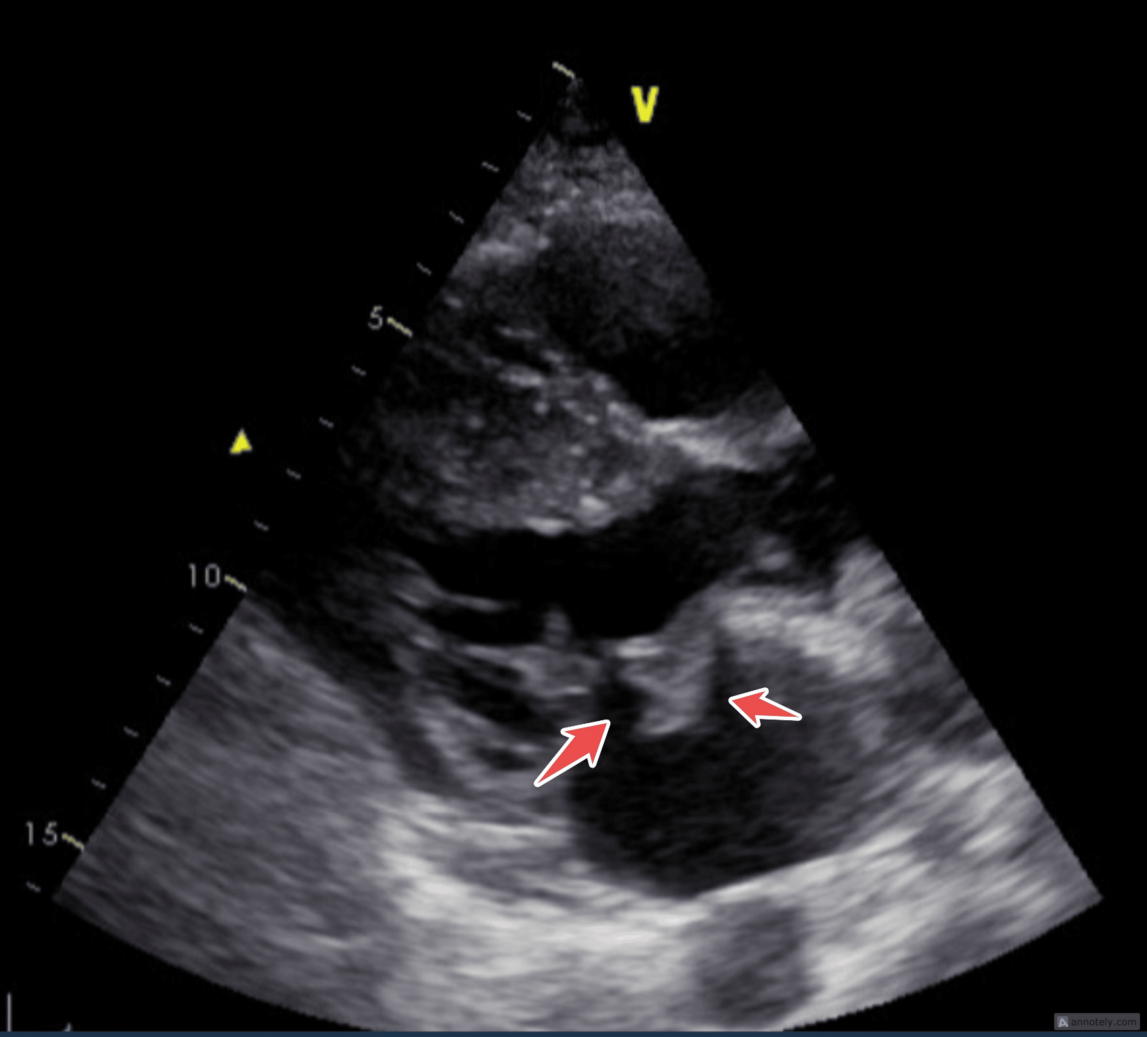
PLAX view showing mitral valve prolapse with vegetation, highlighted by arrows. PLAX: Parasternal Long Axis.

**Figure 2:**
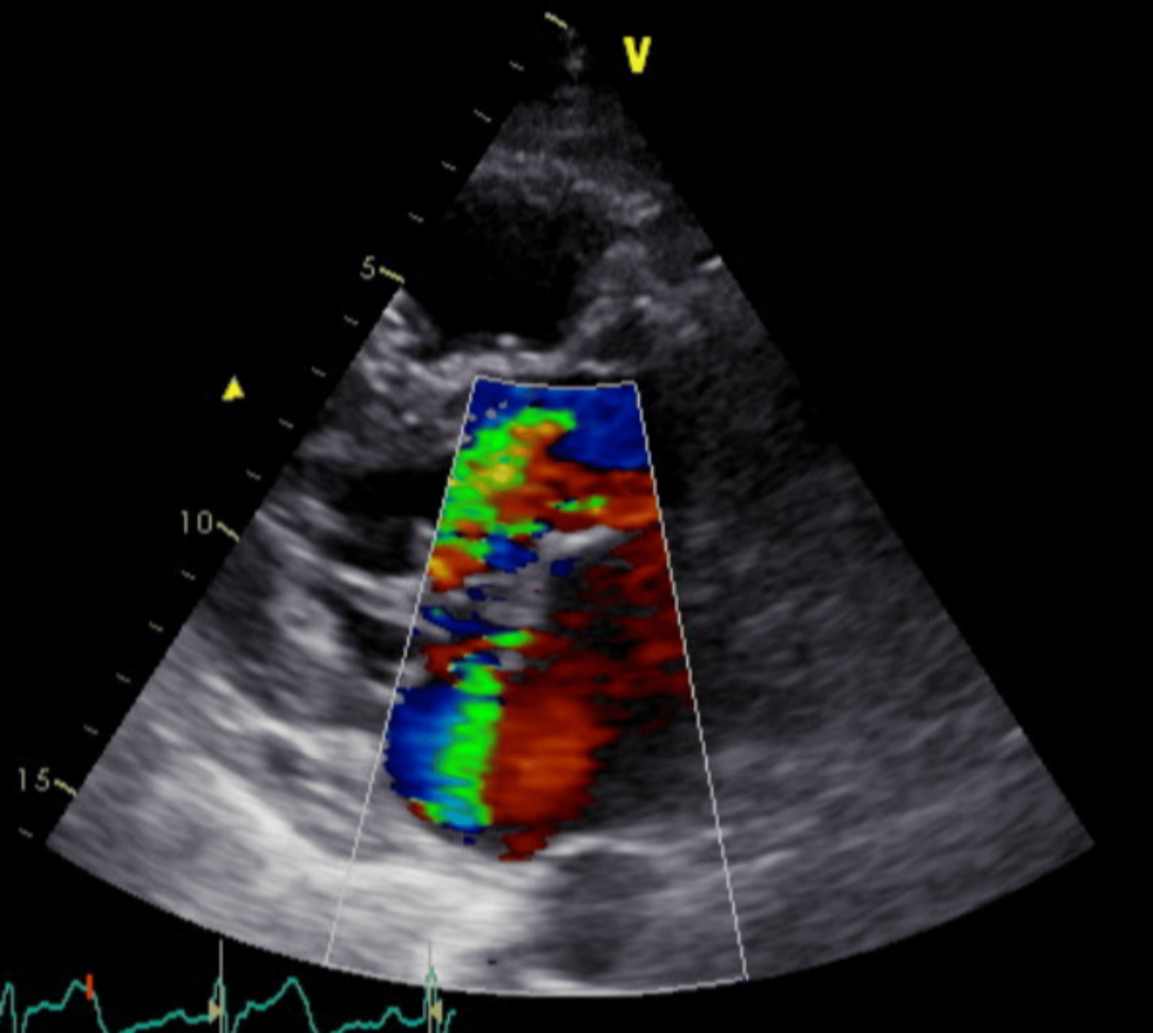
Color Doppler of parasternal long axis showing Mitral Regurgitation

CT scan of the thoracoabdominal and pelvic region was done with no acute findings. Transthoracic echocardiography (TTE) revealed features of Barlow’s disease with a 1.9 cm vegetation on the mitral valve and two jets; one jet from severe mitral regurgitation and the other from the perforated mitral leaflet (Figures 1, 2). The ejection fraction was approximately 50%.

Clinical course and management

Our patient, on the first admission, was started on empiric therapy with intravenous vancomycin (15 mg/kg every 12 hours) plus cefazolin to ensure coverage for both methicillin‑resistant and methicillin‑sensitive S. aureus until susceptibility results were available. Three sets of blood all grew MSSA. Thus, according to the Duke’s criteria of clinical presentation, positive blood cultures and confirmatory TTE results, a diagnosis of infective endocarditis was established. Once MSSA was confirmed the regimen was promptly de‑escalated to cloxacillin 2 g every four hours, maintaining synergistic gentamicin for the first three days.

Following this, a more detailed history revealed that the patient had undergone recent dental work several weeks prior with no prior antibiotic use.

The multidisciplinary team originally planned a ‘medical first’ strategy of four weeks of bactericidal therapy because the patient was hemodynamically stable.

In response to antibiotic therapy, inflammatory markers gradually decreased; the CRP reached 56 mg/L and the white blood cell count reached 8×10^9/L.

**Figure 3:**
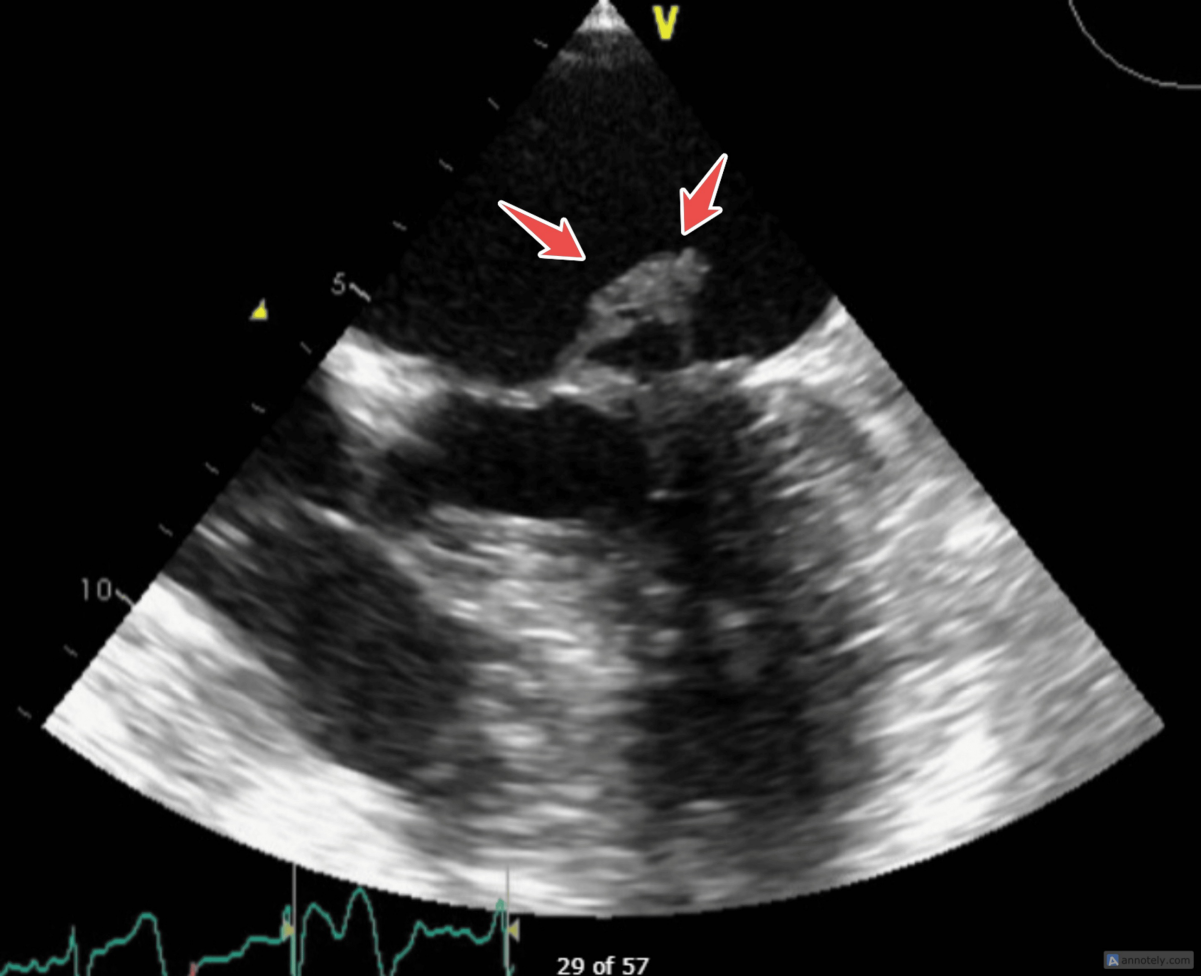
TEE showing vegetation highlighted by arrows. TEE: Transesophageal echocardiogram.

**Figure 4:**
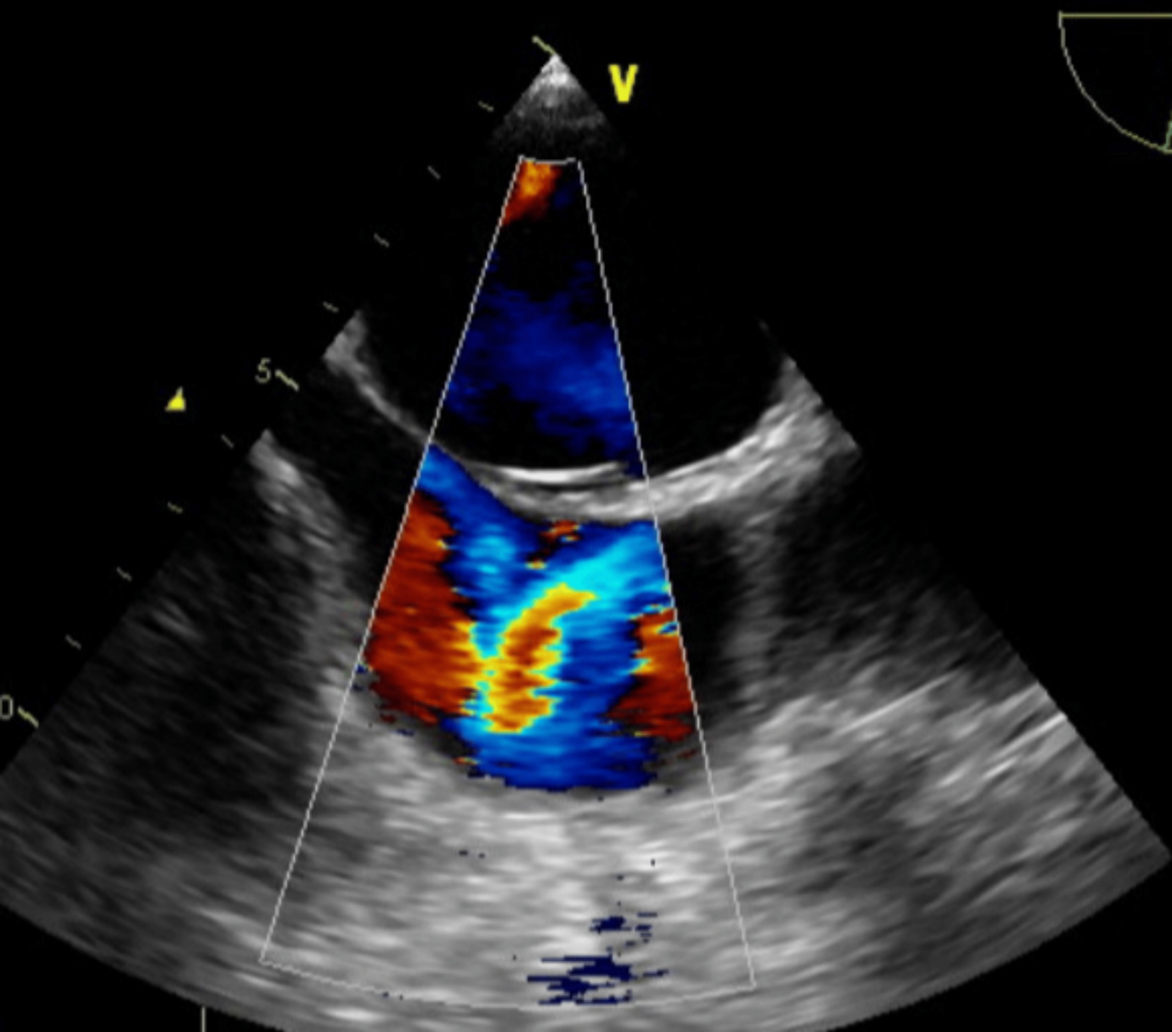
Color doppler on TEE showing severe mitral regurgitation. TEE: transesophageal echocardiogram

**Figure 5:**
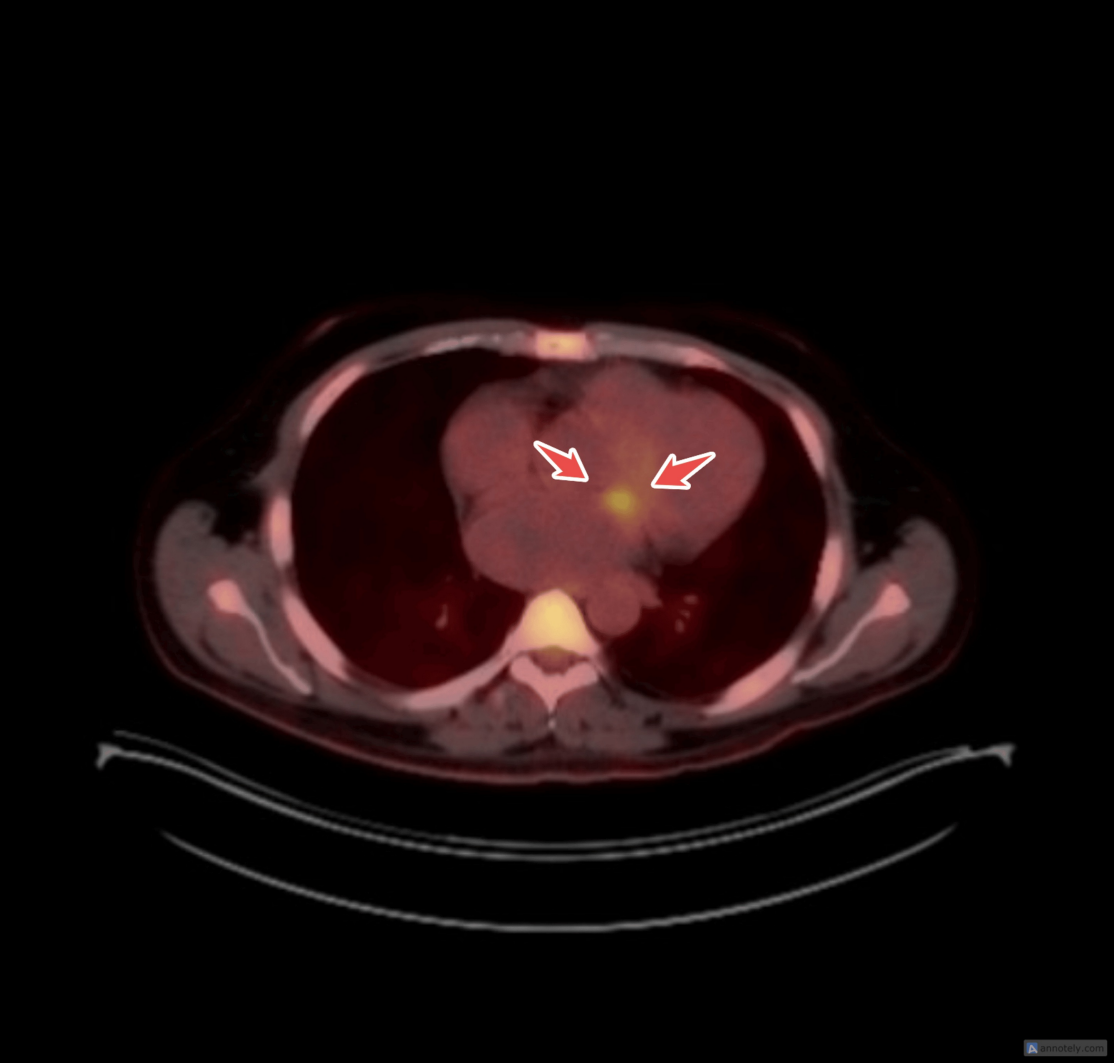
PET scan showing increase metabolic uptake at the mitral valve. PET: positron emission tomography

A follow-up transesophageal echocardiogram (TEE) on day 11 post-admission confirmed persistent vegetation (Figures 3, 4). A positron emission tomography (PET) scan one day later demonstrated an increased uptake in the heart which confirmed the presence of the vegetation (Figure 5). Serial chest X-rays remained normal during this period. 

**Figure 6:**
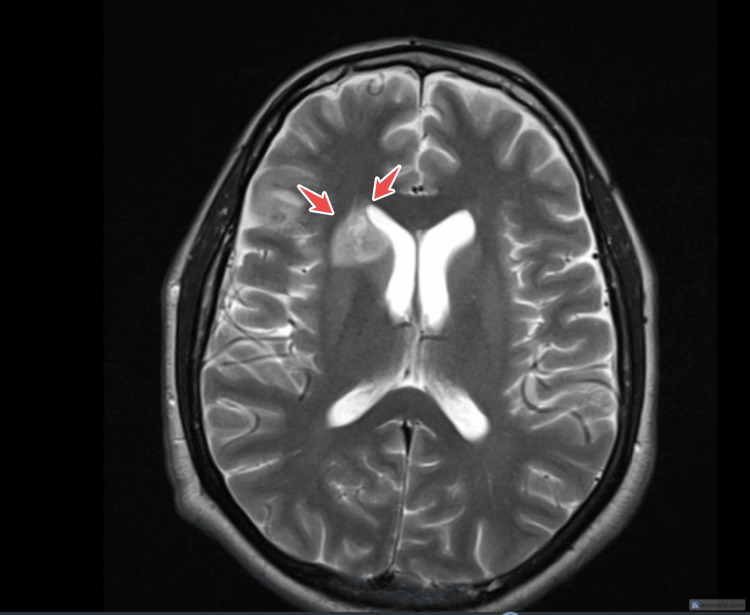
MRI brain showed ischemic stroke. MRI: Magnetic Resonance Imaging.

However, on day 23 post admission, the patient developed left-sided weakness and aphasia. Brain magnetic resonance imaging (MRI) showed evidence of a 1.5 cm mildly restricted diffusion lesion on the right head of caudate nucleus confirming an ischemic stroke (Figure 6). Post‑stroke work‑up included carotid Doppler ultrasound, which was normal, and repeat brain MRI/magnetic resonance angiography (MRA) excluding large‑vessel stenosis, supporting a cardio‑embolic mechanism.

**Figure 7:**
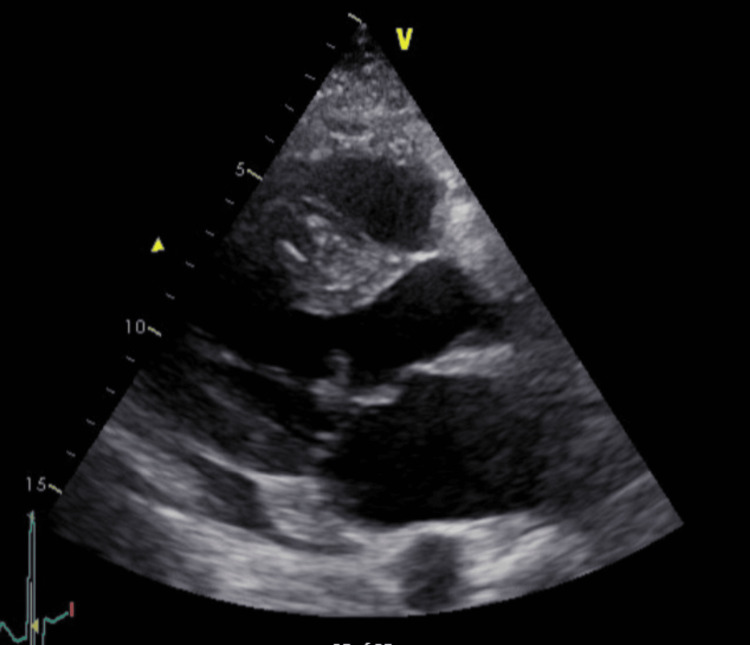
TTE showing resolution of mitral valve vegetation. TTE: transthoracic echocardiogram.

**Figure 8:**
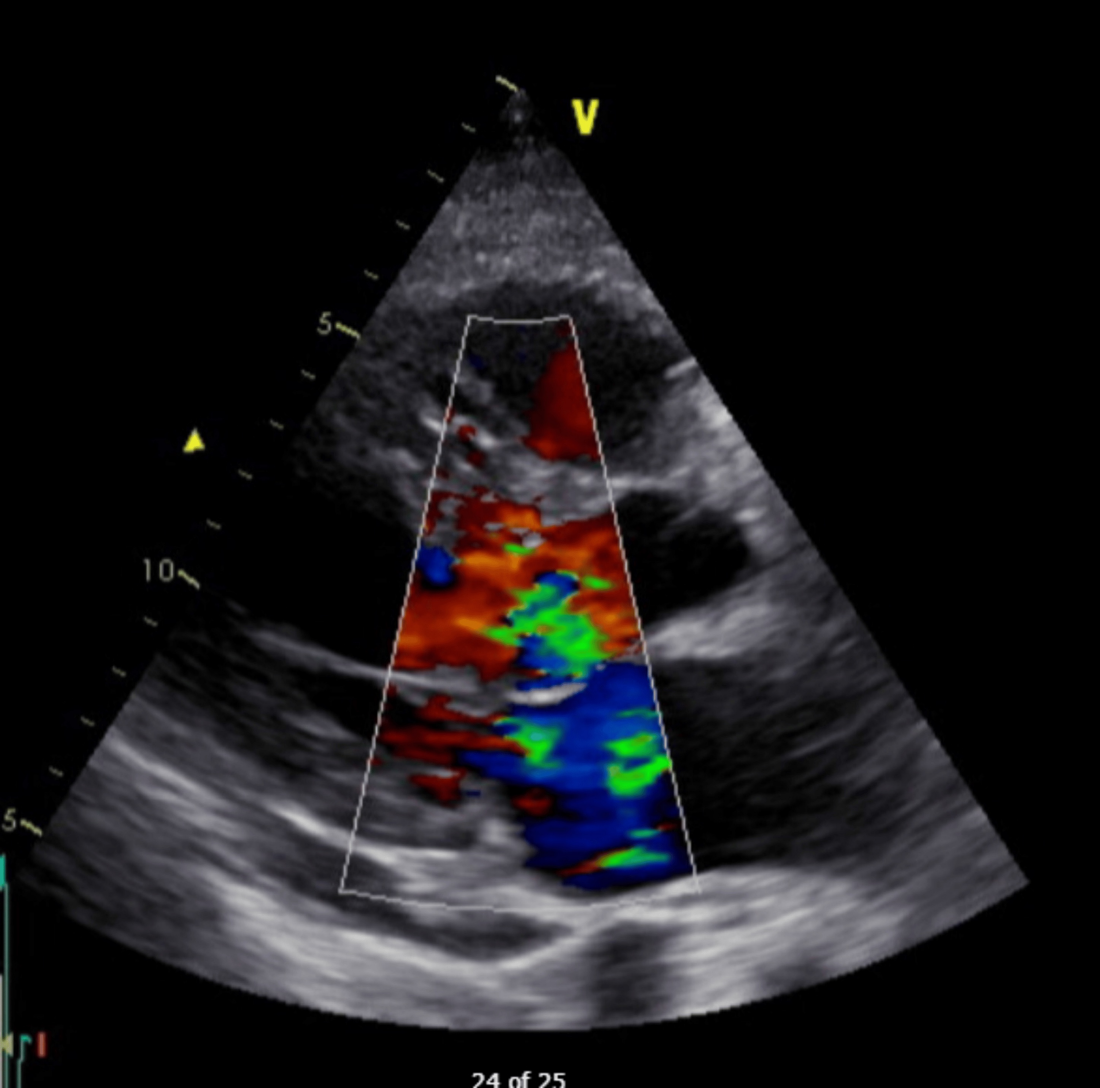
PLAX showing grade II mitral regurgitation. PLAX: Parasternal long axis.

A subsequent TTE could not show the previously visualized vegetation, suggesting embolization to the brain. Therefore, mitral valve repair surgery was done afterwards and the patient was discharged with no complications after TTE was performed and showed an improved mitral regurgitation with grade 2/4 (Figures 7, 8).

The occurrence of a large (23 × 11 mm) mobile vegetation on repeat TEE and a subsequent embolic stroke on day 23 prompted expedited surgical intervention on day 26.

**Figure 9:**
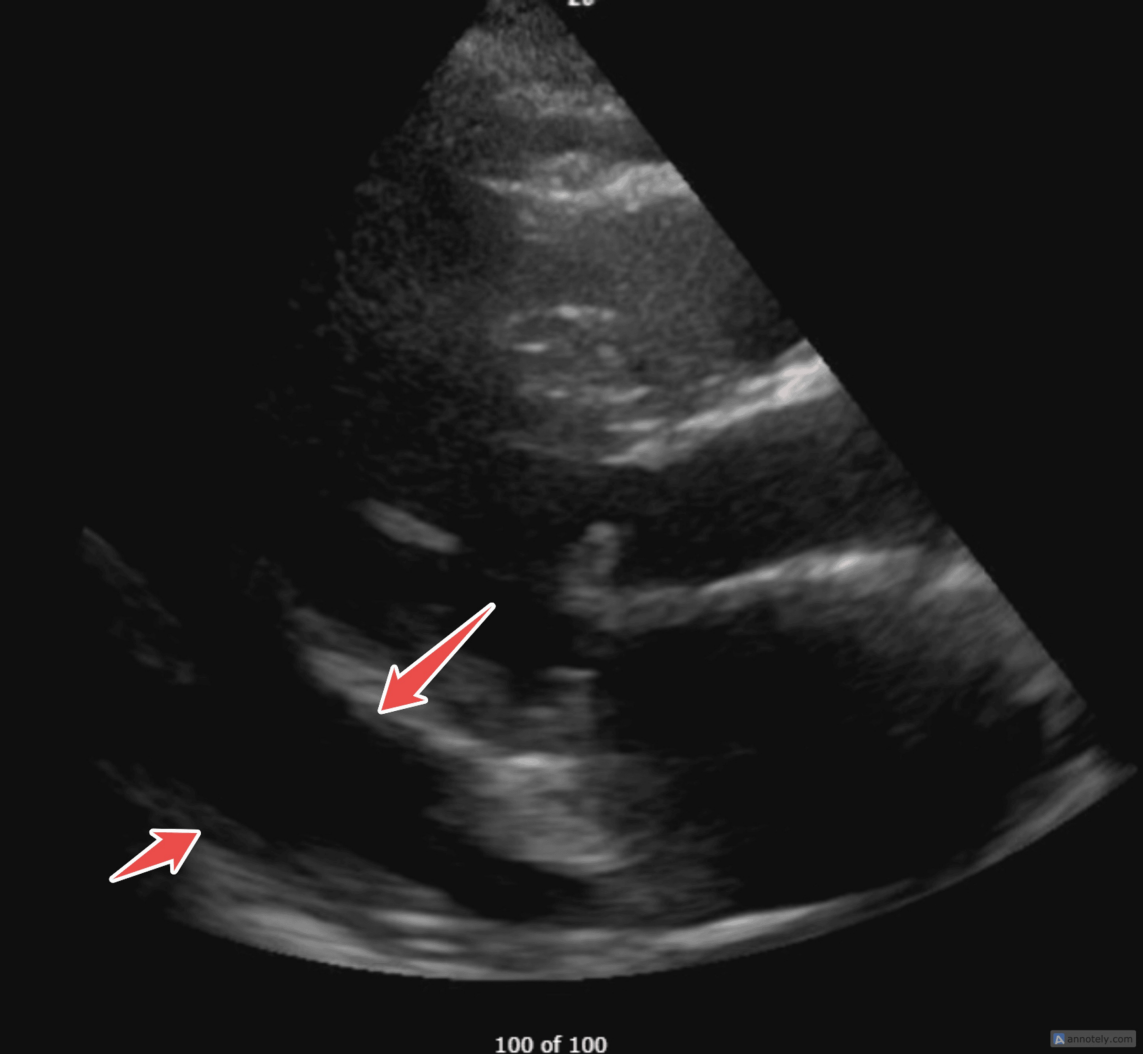
TTE showing severe pericardial effusion TTE: transthoracic echocardiogram

**Figure 10:**
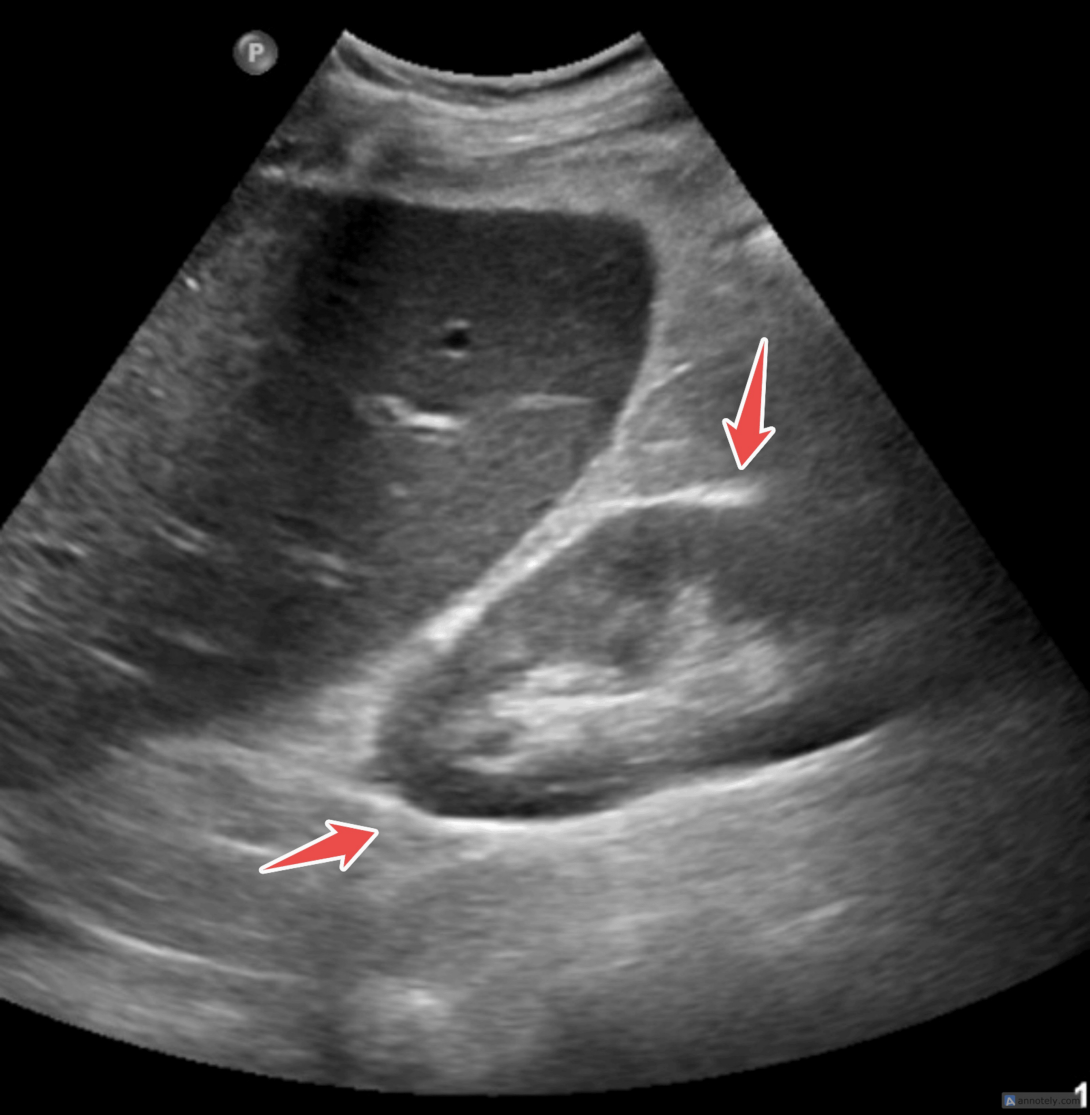
Abdominal ultrasound showing sign of cholecystitis.

Four days later, the patient presented to the emergency department with dyspnea and orthopnea. Pulmonary embolism was suspected, but CT angiography was negative. Bedside echocardiography revealed a 3 cm pericardial effusion on the posterior wall of the left ventricle, with a severe MR with a holosystolic jet and grade 4/4 due to a torn mitral leaflet (Figure 9). Abdominal ultrasound showed gallbladder distention with pericholecystic fluid, consistent with acute cholecystitis (Figure 10). Lab tests showed elevated inflammatory markers (WBC 13 × 10^9/L, CRP 92 mg/L).

After that and while in the emergency department, the patient became hypotensive and was admitted to the ICU (non-intubated) with a working diagnosis of sepsis and therefore, a central venous line was placed. Empiric broad-spectrum antibiotic coverage with intravenous piperacillin-tazobactam (4.5 g every six hours) was initiated for septic cholecystitis, after which serial laboratories showed a steady fall in the leukocyte count from 13 × 10^9/L to 9 × 10^9/L, and in CRP from 92 mg/L to 68 mg/L over the first 48 hours of therapy.

**Figure 11:**
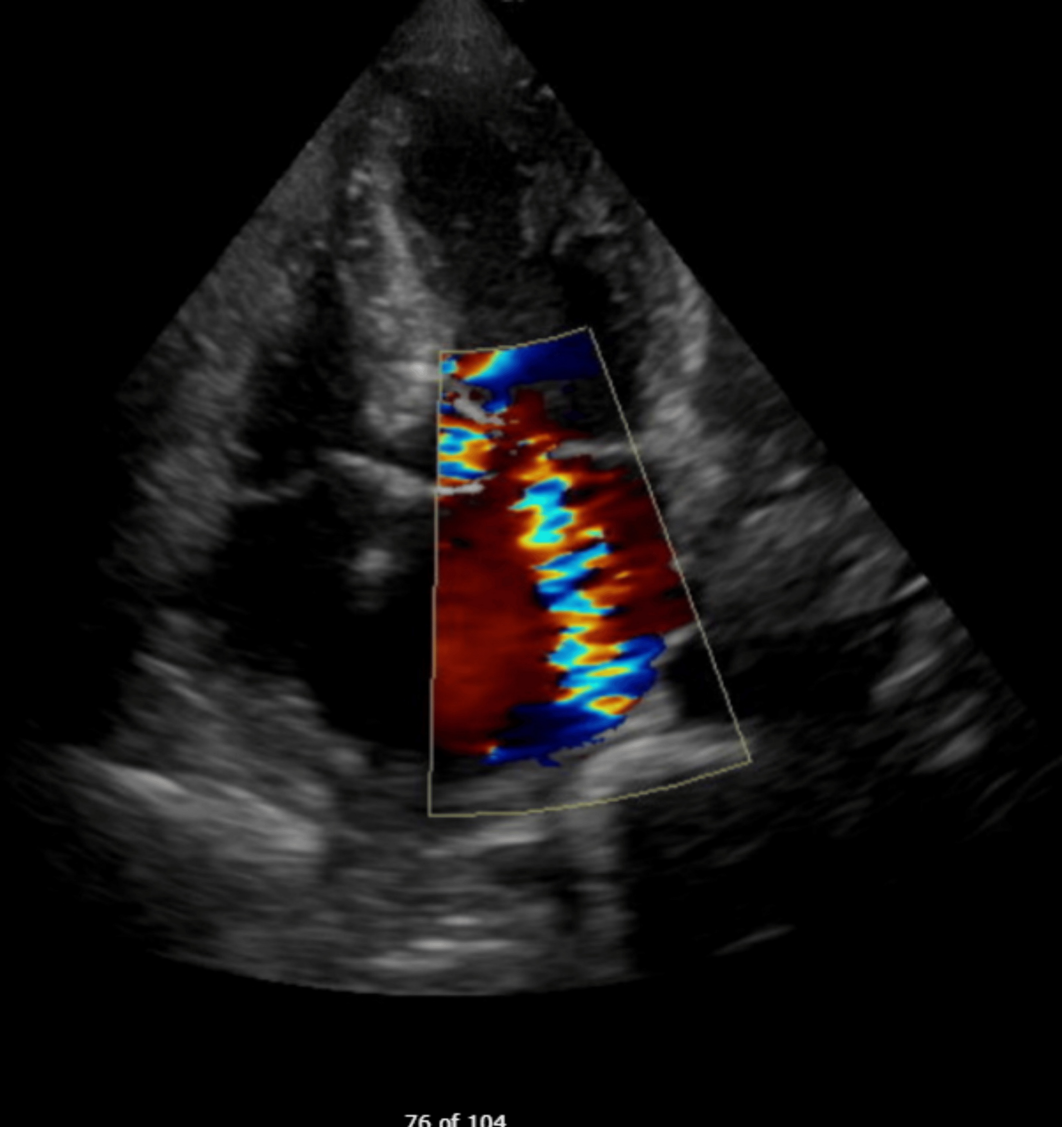
TTE showing regression of pericardial effusion. TTE: transthoracic echocardiogram.

Two days post-second admission ECG revealed atrial flutter and the patient became hemodynamically unstable and required intubation. Repeat TTE showed an improvement in the pericardial effusion but persistent severe mitral regurgitation (Figure 11).

**Figure 12:**
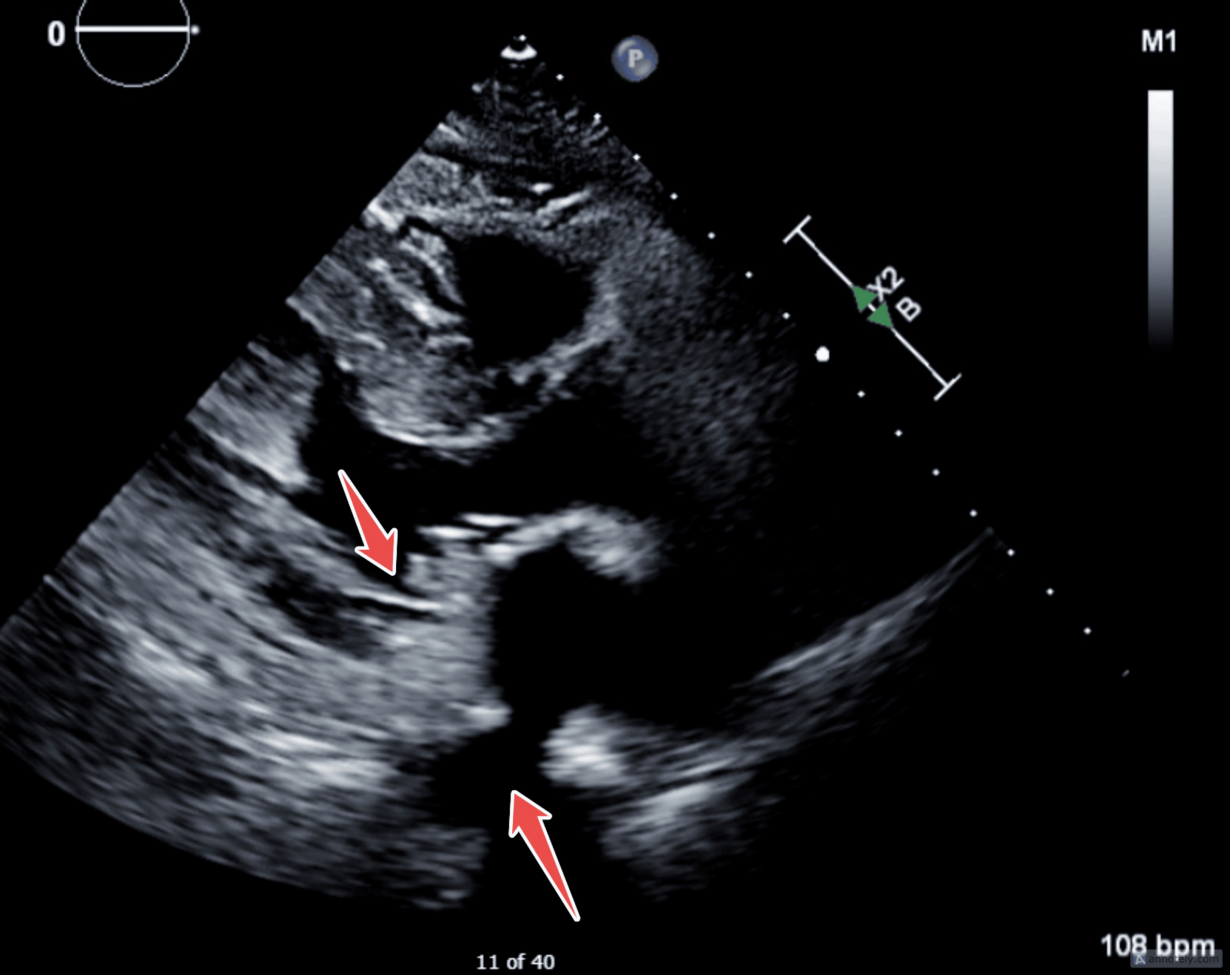
TTE in PLAX view post mechanical mitral valve replacement highlighted in arrows. TTE: transthoracic echocardiogram, PLAX: parasternal long axis

Following all this, the patient subsequently became stable and extubated, then underwent a cholecystectomy, followed days later by mitral valve replacement with a mechanical prosthesis. Acute calculous cholecystitis, confirmed by ultrasound and intra‑operative pathology, was treated surgically and considered an independent event rather than an embolic manifestation. Before discharge the patient and family received structured education on oral hygiene, anticoagulation vigilance and infective endocarditis prophylaxis per 2023 European Society of Cardiology (ESC) guidelines; acenocoumarol was titrated to a target international normalized ratio (INR) of 2.0 - 3.0 with the first outpatient INR check scheduled at 48 hours. WBC count, CRP and procalcitonin normalised by postoperative day 12 and remained stable during the three‑week clinic follow‑up, during which TTE documented a competent prosthetic valve and no residual effusion (Figure 12).

## Discussion

The clinical management of IE proves to be challenging when it occurs with structural valvular abnormalities like Barlow’s disease because this condition increases the risk of bacterial colonization and embolic complications. Patients with vegetations exceeding 10 mm size should undergo surgical intervention according to European and American guidelines especially when Staphylococcus aureus causes the infection due to its high virulence and embolic potential [10,11].

Barlow’s disease increases the risk of infective endocarditis because it leads to myxomatous degeneration of the mitral valve which results in redundant leaflet tissue along with annular dilation. The abnormal valve structure along with turbulent blood movement creates optimal conditions for bacterial cells to attach and multiply [12]. The most frequent pathogens responsible for IE after dental procedures are Streptococcus species, yet MSSA infections occur less frequently, especially in patients who have inadequate dental care or undiagnosed structural heart defects [13]. The latest discussions about valvulopathy prophylaxis strategies confirm the necessity of antibiotic protection for individuals with high-risk profiles even when endocarditis has not occurred before [14].

The embolic complications, which include ischemic stroke and acute cholecystitis along with pericardial effusion, demonstrate how IE affects the entire body and requires teamwork from multiple specialists. The occurrence of embolic phenomena affects 50% of patients with infective endocarditis and leads to elevated mortality rates [15]. TTE and TEE and PET/CT imaging played an essential role in determining vegetation persistence and systemic disease spread. PET imaging demonstrates high value as a diagnostic method to detect metabolically active lesions in prosthetic valve endocarditis and complex native valve infections [16].

Large (≥10 mm) vegetations, particularly those caused by highly virulent pathogens such as MSSA, possess greater kinetic energy and are prone to fragmentation [16]. We believe this combination of virulence and vegetation size explains the patient’s neurologic embolism despite bactericidal therapy. The absence of radiological or histopathological evidence of septic emboli in this case of acute cholecystitis makes it an incidental finding rather than an embolic manifestation. Although 18F‑FDG PET/CT adds incremental diagnostic value in prosthetic‑valve IE, its sensitivity in native‑valve disease is modest (~43%) and current guidelines recommend its use only in complex or inconclusive cases. Our utilisation was therefore supportive and not definitive. Active participation of a Multidisciplinary Endocarditis Team (MDET) could have accelerated the shift from medical to surgical management when the vegetation persisted, potentially preventing the stroke and early repair failure. The 2023 American Heart Association (AHA)/American College of Cardiology (ACC) and ESC guidelines reserve antibiotic prophylaxis for patients with prosthetic valves, prior IE or selected congenital heart disease. Therefore, we acknowledge that advocating prophylaxis for every patient with Barlow’s morphology exceeds current evidence. Instead, we recommend meticulous dental hygiene and risk‑based prophylaxis. Finally, early valve replacement, as opposed to conservative repair, may offer better durability in markedly myxomatous valves and might have averted the postoperative recurrence seen in our patient.

The post-repair severe mitral regurgitation that required valve replacement demonstrates a significant characteristic of Barlow’s disease. The extensive nature of leaflet redundancy and annular dilation in these cases suggests that valve repair may not last so early mechanical valve replacement should be considered [17]. The long-term benefits of mechanical prostheses in treating complex myxomatous valve disease are supported by various research studies [18].

Early, guideline‑directed surgical intervention and close MDET oversight are crucial in Barlow‑associated IE to minimise embolic events and ensure durable valve therapy; risk‑stratified antibiotic prophylaxis and optimisation of dental hygiene remain cornerstones of prevention.

This case demonstrates why MDETs should be implemented as recommended by the European Society of Cardiology. These teams, which combine cardiology, infectious diseases, cardiothoracic surgery, radiology and neurology expertise, help patients survive better by providing timely coordinated care [19]. This patient’s clinical progression from initial medical treatment to delayed surgery followed by embolic complications and concluding with mechanical valve replacement and cholecystectomy demonstrates how MDET involvement could have improved early decision-making throughout their treatment.

## Conclusions

This scenario underscores the challenges of dealing with a case of endocarditis coupled with septic embolization and structural valve issues. It stresses the significance of collaboration among medical specialties, early evaluation of opting for valve replacement instead of repair and thorough examination of antibiotic prevention methods for high-risk patients with structural heart disease. Healthcare providers are advised to contemplate administering antibiotics before dental treatments for individuals with Barlow’s disease to lower the chances of infective endocarditis occurrence.
